# The Causal Role of Consciousness in Psychedelic Therapy for Treatment-Resistant Depression: Hypothesis and Proposal

**DOI:** 10.1021/acsptsci.5c00445

**Published:** 2025-07-16

**Authors:** Tobías Fernández-Borkel, Lucas F. Borkel, Jaime Rojas-Hernández, Elisa Hernández-Álvarez, Domingo J. Quintana-Hernández, Ludovica G. Ponti, Luis Alberto Henríquez-Hernández

**Affiliations:** 1 Center for MR Research, University Children’s Hospital Zurich, 8032 Zurich, Switzerland; 2 Department of Forensic and Neurodevelopmental Sciences, Institute of Psychiatry, Psychology & Neuroscience, King’s College, London WC2R 2LS, U.K.; 3 Asociación Científica Psicodélica, 35412 Canary Islands, Spain; 4 Health Sciences Faculty, 16750 Universidad de Las Palmas de Gran Canaria, 35016 Las Palmas de Gran Canaria, Spain; 5 Asociación Canaria para el Desarrollo de la Salud a través de la Atención, 35007 Las Palmas de Gran Canaria, Spain; 7 Universidad del Atlántico Medio, Faculty of Psychology, 35017 Las Palmas de Gran Canaria, Spain; 8 Department of Biomedical Engineering, 27209 University Basel, 4001 Basel, Switzerland; 9 Unit of Toxicology, Clinical Sciences Department, Universidad de Las Palmas de Gran Canaria, 35016 Las Palmas de Gran Canaria, Spain

**Keywords:** psychedelics, consciousness, anesthesia, TRD, psilocybin, clinical trial

## Abstract

The therapeutic potential of psychedelic substances, particularly psilocybin, for treatment-resistant depression (TRD) has garnered considerable attention. However, the necessity of subjective psychedelic experiences for therapeutic efficacy remains unclear, creating a critical gap in the field. To determine whether subjective psychedelic experiences induced by psilocybin are required for its antidepressant effects or whether these effects are mediated solely by neurobiological actions independent of consciousness. We propose a randomized controlled trial with three groups: (P) Psilocybin (25 mg oral dose with guided therapeutic integration), (P+A) Psilocybin under propofol-induced general anesthesia (eliminating subjective experiences), and (X+A) Propofol-induced anesthesia with placebo (with no psilocybin). Clinical assessments of depression and anxiety, combined with fMRI-based brain connectivity analysis (including fractal complexity, brain entropy, and network dynamics), will be conducted at baseline, postintervention, and during follow-ups. The proposed study protocol expects distinct therapeutic outcomes across groups. Superior improvements in depression and anxiety symptoms are anticipated in the conscious psilocybin group (P) compared to both anesthetized groups (P+A) and (X+A). Additionally, increased brain connectivity measures in fractal complexity and entropy are hypothesized to correlate positively with therapeutic improvements, particularly pronounced in the conscious condition. Isolating subjective experiences through anesthesia, aims to determine whether conscious psychedelic experiences play a causal role in therapeutic outcomes. Results have significant implications for clinical protocols, treatment guidelines, and the broader theoretical understanding of consciousness and its relationship to mental health.

## Graphical abstract

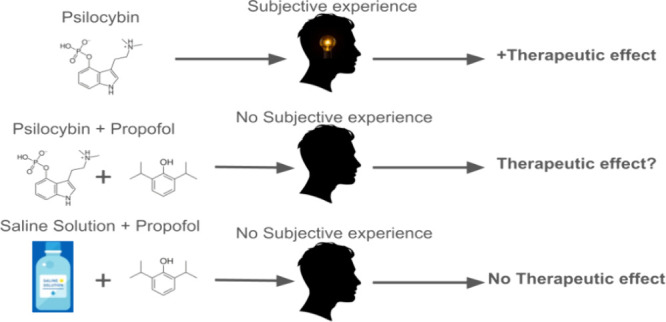

## 1 Background

In the past decades, serotonergic psychedelic substances, such as LSD, psilocybin, and mescaline have attracted substantial interest for their medical applications^1,2^ and their capacity to explore the phenomenology of altered states of consciousness^3,4^ Psychedelics induce alterations in sensory perception, including visual distortions, complex imagery, shifts in self-perception, and emotional extremes, such as psychotic episodes.^5^ Psychedelic experiences often evoke deeply personal, spiritual, or religious experiences,^6^ a phenomenon which has made them integral to religious practices in various cultures.^7^

Neuroimaging studies using functional magnetic resonance imaging (fMRI) and magnetoencephalography (MEG) have linked subjective qualities of the psychedelic state to increased brain activity entropy, as proposed by the Entropic Brain Hypothesis (EBH).^8,9^ The EBH posits that waking consciousness operates near a critical zone, consisting of a balance between order and disorder. In contrast, psychedelics increase entropy, “pushing” the brain to this critical threshold.^8^ Criticality represents a phase transition between two distinct states: the subcritical state, characterized by rigidity, low entropy, and order, and the supercritical state, marked by high entropy, flexibility, and disorganization.^10^ This hypothesis aligns with broader theories indicating that critical systems possess optimal information-processing capacities, such as memory, communication, and dynamic range.^11–13^ Approaching the critical point often produces stereotyped patterns such as fractal structures, which can serve as indirect indicators of criticality. This might aid in understanding the functional and experiential effects of psychedelics and their potential role in enhancing cognitive and sensory flexibility. Recent research in neuroimaging and gradient-mapping techniques have revealed that psilocybin significantly disrupts hierarchical cortical organization, including reduced differentiation between unimodal (sensory) and trans-modal (default mode and associative) networks, promoting a reduction of the macro-scale cortical connectivity gradient.^14^ These findings align with the Relaxed beliefs under psychedelics model (REBUS), which posits that psychedelics destabilize hierarchical predictions encoded in high-level cortical networks, enhancing the sensitivity to bottom-up sensory input.^15^

### 1.1 Psychedelics as Antidepressants

Psilocybin, a partial agonist at serotonin 5-HT2A receptors, has demonstrated therapeutic potential for treatment-resistant depression, showing no dissociative and psychedelic effects.^16^ Stimulation of these receptors promotes synaptic plasticity, increases dendritic spine density, and induces changes in large-scale brain network connectivity.^17^ Carhart-Harris observed that psilocybin significantly alleviated depressive symptoms in patients with Treatment-Resistant Depression (TRD), with these effects persisting for up to six months.^3^ These therapeutic effects have been associated with a hypothesized “resetting” of dysfunctional brain networks, particularly the default mode network (DMN), implicated in self-referential thinking and ruminative thought processes.^18^

Recent studies have shown that psilocybin disrupts hyperconnectivity within the DMN and facilitates increased communication between traditionally segregated networks, such as the salience network (SN) and frontoparietal control network (FCN).^19^ These changes in brain connectivity have been linked with increased emotional flexibility, potentially crucial for the recovery from depressive symptoms.^3^ These neural effects are supported by changes in neuroplasticity, including an increased dendritic spine density and neurogenesis.^17^ Recent studies by Varley have revealed that other psychedelic substances like *N*,*N*-dipropyltryptamine (DPT) alters neural information dynamics, including increased entropy of firing activity and a proliferation of weak, low-strength neural connections.^10^ This enhanced neuroplasticity could potentially contribute to broader reorganizational changes in those neural networks underlying therapeutic effects of psychedelics.

### 1.2 The Role of Conscious Experience

The therapeutic efficacy of psychedelic compounds, such as psilocybin, has been linked to the subjective experience they induce. Roseman proposed a strong correlation between the extent of emotional breakthrough during psilocybin therapy and reductions in depressive symptoms.^20^ Amygdala responses were increased 1 day subsequent to the psilocybin administration, being also predictive for positive clinical outcomes of depression.^20^ Subjective experience of psychedelics has been proposed to be required to achieve their therapeutic outcomes^21^ and other recent findings have also shown that set and setting variables, including motivation of use, location and social company, have a crucial role regarding psychological (e.g., subjectively experienced well-being) and psychopathological outcomes.^22^

The only known neuroplastic process which allows for the update and transformation of the emotional learnings underlying symptoms is memory reconsolidation.^23^ The occurrence of this process necessitates the reactivation of the pertinent memory in the patient’s consciousness, thereby establishing a period of plasticity during which these learnings can be modified. The therapeutic potential of psychedelics, which have been shown to facilitate a state of heightened suggestibility and intensified access to implicit or nonconscious emotional content,^24^ is largely contingent on the conscious activation of these learnings. However, if the reactivation process is impeded, as it might be by the use of general anesthesia, then reconsolidation cannot occur and the emotional learnings causing the symptoms cannot be modified.

In this regard, a comparative study of the effects of psychedelics under general anesthesia versus their administration in a conscious state could provide pivotal evidence regarding the role of consciousness in the therapeutic process. If the specific benefits of psychedelics depend on reconsolidation, only those patients who remain conscious during the experience should be able to exhibit a lasting and substantial change in their symptoms. Conversely, if the anesthetized group exhibits analogous improvements, this would imply that the therapeutic effects of psychedelics function through nonspecific mechanisms, such as neurochemical changes or global modifications in brain plasticity, without necessitating phenomenological consciousness. This potential research could help determine whether subjective experience is a causally indispensable component for deep therapeutic change or whether, on the contrary, the effects of psychedelics can be explained without invoking consciousness as a mediating factor.

Supporting the importance of the reconsolidation process, several recent studies provide valuable insights into how psychoactive substances can directly affect memory reconsolidation. Hake demonstrated that 3,4-methylenedioxymethamphetamine (MDMA) administered during the reconsolidation phase led to lasting reductions in conditioned fear responses, highlighting its direct role in memory modulation.^25^ Similarly, Corlett found that ketamine administration during memory reconsolidation significantly impacted memory strength by altering prediction error signaling, further supporting the importance of reconsolidation processes.^26^ Additionally, Daneluz showed that Ayahuasca impairs fear memory reconsolidation in animal models, further supporting the notion that certain psychedelic substances can effectively modify emotional memories during specific reconsolidation windows.^27^ These studies collectively underline the critical interaction between conscious memory reactivation, psychedelic treatment, and therapeutic outcomes. They suggest that subjective consciousness might indeed be essential, particularly because reconsolidation requires the active recall and processing of emotional content. Therefore, clarifying the precise role of conscious subjective experience remains pivotal for optimizing psychedelic therapy protocols and deepening our understanding of the underlying neuropsychological and therapeutic mechanisms.

Taken together, these findings support the notion of subjective experience, induced by psychedelics, as a potential requirement to obtain therapeutic benefits,^21^ besides the underlying neurobiological mechanisms. To test this hypothesis, it has been suggested that the only definitive way to disprove the importance of subjective psychedelic effects is the administration of a psychedelic to fully unconscious individuals who subsequently report a lack of memories about the psychedelic experience.^21^

In line with this hypothesis, a recently designed proposal,^28^ currently in progress, explicitly examines whether the psychoactive effect is necessary for the antidepressant efficacy of psilocybin. The trial compares the effects of psilocybin administered alone versus psilocybin combined with risperidone, a serotonin 2A (5-HT2A) receptor antagonist known to block psychedelic effects. Although risperidone administration may significantly reduce or eliminate typical psychedelic phenomena, participants remain conscious during the treatment, potentially retaining some form of subjective experience. Thus, while this study provides crucial insights into the specific role of 5-HT2A receptor-mediated psychedelic effects, we propose to use general anesthesia to fully suppress consciousness. This approach could offer a stronger and more definitive test of the necessity of subjective experience for therapeutic benefits. Such an experiment could delineate the interplay between psychedelic’s therapeutic efficacy and states of consciousness and share light on the question of whether the therapeutic benefits of these substances are contingent upon the subjective experiences they elicit. Determining whether conscious experience mediates neural connectivity and directly influences the antidepressant effects of psychedelics is a question of relevance that, to our knowledge, remains unexplored.

## 2 The Role of Subjective Experience in Psychedelic Assisted Therapy: An Experimental Proposal

### 2.1 Aims and Rationale

This experimental design seeks to clarify the critical question of whether the subjective psychedelic experience itself is essential to the antidepressant effects of psilocybin, or whether the therapeutic effects derive primarily from direct neurobiological alterations induced by the substance (Figure 1). Psilocybin administration during general anesthesia could provide a unique and controlled scenario in which the neurochemical effects can be decoupled from the subjective phenomenology typically associated with psychedelic states. Propofol was selected due to its capacity to reliably induce unconsciousness, characterized by a suppression of subjective experience and memory formation, without severely affecting fundamental neurobiological pathways associated with serotonin receptor modulation.^29,30^ Unlike other sedatives or natural states like deep sleep or sedation, propofol reliably prevents the conscious recall of events and significantly diminishes cortical activity associated with consciousness, ensuring participants remain unconscious throughout the psilocybin-induced state. This design allows for a clear dissociation between purely neurochemical effects and subjective experience-dependent processes.

**Figure 1:**
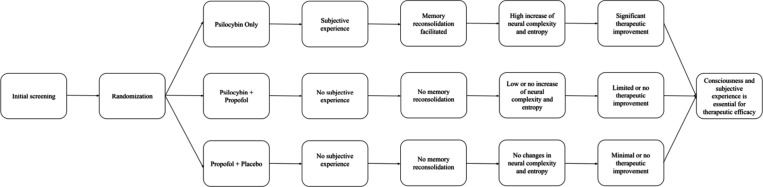
Hypothesized relationship between consciousness (subjective experience), neural connectivity, and therapeutic outcomes in psychedelic therapy.

It is important to acknowledge that propofol itself influences brain connectivity, particularly affecting cortical synchronization and functional connectivity patterns. Nevertheless, previous research has clearly characterized propofol’s neural signatures,^29^ which differ distinctly from the connectivity profiles typically associated with psychedelic states, such as increased entropy and fractal complexity. By carefully measuring and comparing connectivity changes across both conscious and unconscious conditions, this study can reliably differentiate between propofol-induced alterations and those specifically related to psilocybin administration. Moreover, since propofol’s mechanisms involve modulation of GABA-ergic neurotransmission^31^ without directly acting on serotonergic pathways, no pharmacological interaction is expected. This reduces confounding factors and enables a clearer interpretation of the role of subjective experience. Importantly, the anesthetic effects of propofol are short-lived, reversible,^32^ and clinically well-understood, mitigating ethical concerns regarding prolonged unconsciousness.

### 2.2 Hypotheses

1. Absence of conscious experience, due to general anesthesia, may attenuate psilocybin-induced antidepressant effects.
2. Subjective experiences mediate the relationship between psilocybin-induced neural connectivity changes and clinical outcomes.

## 3 Methods

This study would involve a randomized design to investigate the role of consciousness in the antidepressant effects of psilocybin by comparing three variants of treatment: psilocybin only (P) versus psilocybin with general anesthesia (P+A) versus general anesthesia with placebo (X+A) (see Table 1).

**Table 1:** Time Schedule of Enrollment, Interventions, and Assessments^a^

	week −1	baseline (week 0)	day 1 postintervention	follow-up 1 (week 1)	follow-up 2 (week 3)	follow-up 3 (week 6)	follow-up 4 (month 3)	follow-up 5 (month 6)
**Enrollment**	√	√						
Eligibity screen	√							
Informed consent	√							
Randomization (p, p+a, x+a)	√							
**Interventions**								
psilocybin + therapy (p)		√						
psilocybin + anesthesia (p+a)		√						
placebo + anesthesia (x+a)		√						
therapeutic integration (for p group)		√	√	√	√	√		
**Assessments**								
Depression (BDI)		√	√	√	√	√	√	√
anxiety (STAI)		√	√	√	√	√	√	√
fMRI (Connectivity, entropy, fractal)	√	√	√	√	√	√		
**Safety Monitoring**		√	√	√	√	√	√	√
Adverse events recording		√	√	√	√	√	√	√

a Note: Dose of psilocybin = 25 mg.

### 3.1 Sample

This study recruits adult participants, clinically diagnosed with moderate-to-severe Treatment-Resistant Depression (TRD), as assessed by the Beck Depression Inventory (BDI),^33^ and unresponsive to standard antidepressant treatments and psychotherapy. Baseline clinical measures of depression and anxiety will be obtained as detailed in Section 3.5.1 and matched across participants to ensure comparability between treatment conditions.

### 3.2 Sample Size

We aim to select a total sample size of participants (*n* = 9, *n* = 3 per group). This size balances technical, and logistical considerations, particularly given the complexity of combining psychedelic administration, general anesthesia, and advanced fMRI and computational analysis. While not statistically powered for hypothesis testing, the *n* = 9 allows to detect trends and feasibility parameters to inform future trials with larger cohorts.

### 3.3 Exclusion Criteria

This study includes participants diagnosed by the Beck Depression Inventory (BDI)^33^ with moderate-to-severe depression and unresponsive to standard antidepressants and psychotherapy (TRD). Baseline clinical measures in depression severity and anxiety levels, are matched across participants to ensure comparability between treatment conditions.

Participants must be psychedelic-naive. Individuals exhibiting clinical incompatibilities, such as a history of psychotic episodes or symptoms, are excluded from the study. Participants with a personal history of psychosis, including major depressive disorder (MDD) with psychotic features and those diagnosed with comorbid borderline personality disorder are not eligible. Individuals with first-degree relatives diagnosed with schizophrenia, schizoaffective disorder, or bipolar disorder are also excluded to mitigate the risk of adverse psychiatric outcomes.^34^ Classic psychedelics present cardiovascular risks, including arrhythmias, hypertension, and ischemia.^35^ Given the limited evidence on the safety protocols of psychedelic studies for individuals with cardiovascular conditions,^36^ participants with cardiovascular diseases are excluded from the study.

Regarding the use of propofol, the main exclusion criteria are individuals with significant cognitive impairment, such as severe dementia and Glasgow Coma Scale (GCS) score of less than or equal to 12, and treatment with butorphanol or similar analgesic agents a month before.^37^

### 3.4 Measurements

- Neuroimaging: Resting-state fMRI to assess functional connectivity and network dynamics, with a focus on the DMN and SN.
- Clinical measurements: Depression severity levels are assessed using the BDI.^33^ Levels of anxiety are assessed using the State-Trait Anxiety Inventory (STAI).^38^

### 3.5 Study Design

#### 3.5.1 Intervention

- Psilocybin Only (P): Participants administered with an oral dose of psilocybin in 25 mg,^39,40^ followed by guided therapy sessions to facilitate the integration of the psychedelic experience at 1 day, 1 week, 3 weeks and 6 weeks after the dosing session.^41^
- Psilocybin+anesthesia (P+A): Participants receive an oral dose of 25 mg of psilocybin. Unconscious state duration must be adjusted to psilocybin pharmacokinetics (e.g., from four up to 6 h).^42,43^ Therefore, propofol is administered 5 min subsequent to the psilocybin administration, to prevent a state of consciousness during the effects of psilocybin.
- Anesthesia+Placebo (X+A): Participants undergo general anesthesia through the administration of propofol and receive an inert placebo (i.e., saline oral solution) instead of psilocybin. To match group conditions from (P+A), propofol is administered 5 min subsequent to the saline administration.
- Blinding: (P+A) and (X+A) groups are assigned under a double-blinded protocol to prevent confounding factors and the effects of participants’ expectations in clinical and neuroimaging outcomes.

Participants are stratified based on depression severity according to the BDI and anxiety levels following the STAI. Additionally, they are matched by age and sex to reduce potential confounding effects. The randomized design is constrained to ensure that the three treatment groups, (P), (P+A), (X+A), maintain comparable baseline clinical measurements. This minimizes variability between groups, increasing the reliability of findings and allowing for a more precise assessment of psilocybin’s effects on clinical outcomes and subsequent changes in brain connectivity.

#### 3.5.2 Allocation

We also use a block randomization method, with fixed block sizes of three participants, to preserve equal group sizes. The sequence is generated using the blockrand package in R (version 4.3.3). In order to minimize the observer bias, randomized allocation is created and stored by an independent researcher at Universidad de las Palmas de Gran Canaria—not involved in participant recruitment, outcome assessment, or further analysis.

#### 3.5.3 Timeline of Measurements

- Baseline: To ensure that pre-existing psychopathology does not interfere with the clinical outcomes of the intervention, clinical scores for depression and anxiety are assessed several days prior to the intervention. It allows for a more accurate baseline measurement, minimizing the potential influence of the participants’ expectations and the levels of anxiety about the upcoming intervention. FMRI screenings are conducted 1 week prior to the intervention.
- During intervention: Prolonged fMRI screenings and no clinical measurements.
- Postintervention: Repeated fMRI screenings and clinical measures repeated at 1 day, 1 week, 3 weeks and 6 weeks postintervention, coinciding with the guided therapy sessions.
- Follow-Up: Assessment of clinical measurements repeated at 3 and 6 months.

#### 3.5.4 Spatial and Temporal Fractal Dimension Analysis

The spatial and temporal Fractal Dimension Analysis follows the methodology of previous studies by Varley.^10^ The Compact Box-Burning (CBB) algorithm^44^ is used to estimate the fractal dimension of functional connectivity networks derived from resting-state fMRI data by capturing the distribution of network connections across scales, therefore enabling the identification of criticality-associated fractal structures. The analysis focuses on the DMN, SN, and sensory-motor regions to determine whether psilocybin increases the fractal complexity of connectivity. Furthermore, the Higuchi fractal dimension algorithm^45^ is applied to the time-series of fMRI BOLD signals to assess self-similarity and complexity in brain activity patterns over time. Additionally, Lempel–Ziv Complexity (LZC) could differentiate between psilocybin-induced neural reorganization during conscious and anaesthetized states by quantifying the entropy rate and algorithmic complexity of time-series data from EEG and fMRI recordings.^29^ LZC represents the information density in neural signals and complements fractal analyses by offering a measure of information-rich dynamics.

#### 3.5.5 Functional Network Analysis

Connectivity networks are constructed using correlation matrices derived from resting-state fMRI data. Thresholded binarization (95%) creates adjacency matrices, enabling the identification of network topology changes and shoring shifts in integration and segregation within the DMN, SN, and FCN under the two intervention variants.

#### 3.5.6 Comparative Analyses

##### Psilocybin Only (P)

Changes in fractal dimensions and LZC are compared between baseline and post-treatment to assess the extent of neural complexity and entropy increases during conscious psychedelic experiences.

##### Psilocybin+Anesthesia (P+A)

Similar analyses explore whether neural complexity changes persist in the absence of consciousness, addressing the role of subjective experience in psilocybin’s therapeutic effects.

##### Anesthesia+Placebo (X+A)

Similar analyses explore whether neural complexity changes persist in the absence of consciousness, addressing the role of expectancy.

##### Cross-Condition Comparisons

(a) Determine whether the magnitude of clinical improvement and brain connectivity in (P) is greater than in (P+A) and (X+A), as hypothesized, and (b) evaluate whether alterations in neural complexity and entropy are associated with therapeutic outcomes, based on clinical assessments of psychopathological traits, including depression and anxiety severity.

#### 3.5.7 Expected Outcomes

- (P) group shows better therapeutic outcomes in terms of depression and anxiety than (P+A) and (X+A). (P+A) might present better outcomes than (X+A).
- In direct comparison to (P+A) and (X+A), (P) displays higher spatial and temporal fractal dimensions, and LZC, in brain networks after treatment. After treatment, (P+A) shows higher spatial and temporal fractal dimensions, and LZC, in brain networks than (X+A).
- Therapeutic effects have a longer duration for participants in (P) than in (P+A).
- A potential low increase in fractal and entropy measures in (P+A) suggest that neuroplastic and connectivity-enhancing effects of psilocybin do not depend solely on subjective experiences.

#### 3.5.8 Study Setting and Recruitment Strategy

Clinical recruitment and assessments will be conducted at the Universidad de Las Palmas de Gran Canaria, in collaboration with the Asociación Científica Psicodélica (ACP). Statistical analyses of both the clinical and neuroimaging data will be performed at the Health Sciences Faculty, Universidad de Las Palmas de Gran Canaria, Spain. Participant recruitment will be coordinated by the Universidad de Las Palmas de Gran Canaria through its affiliated clinical services. Baseline assessments of depression and anxiety may be conducted in part by the ACP, under supervision and in adherence to standardized clinical protocols. Moreover, recruitment will be supported by regional outreach (Cabildo de Gran Canaria) and collaboration with mental health professionals to ensure timely enrolment and attainment of the target sample size.

Computational processing of neuroimaging data, including image preprocessing, fractal and entropy analyses, and connectivity-based metrics, will be carried out at the Centre for Neuroimaging Sciences, Institute of Psychiatry, Psychology & Neuroscience (IoPPN), King’s College London, United Kingdom.

#### 3.5.9 Ethical Considerations

Administering psychedelics under general anesthesia poses potential risks associated with the anesthesia itself, such as respiratory depression and cardiovascular changes.^46,47^ These risks are mitigated by strict adherence to medical safety protocols and continuous monitoring by anesthesiology specialists. Informed consent procedures must thoroughly explain the unusual experimental conditions and potential risks, ensuring participants are fully aware and voluntarily consent. Ethical approval from an independent review board or ethics committee must be secured before study commencement, ensuring participant safety, autonomy, and informed consent are rigorously upheld.

The study protocol will be submitted for ethical approval to the Commité de Ética de la Investigación/Comité de Ética de la Investigación con Medicamentos (CEI/CEIm) in Las Palmas Province, Spain. No participants will be enrolled before obtaining formal ethical clearance.

Details regarding data monitoring and auditing procedures will be fully outlined in the documentation submitted for ethical approval to the Commité de Ética de la Investigación/Comité de Ética de la Investigación con Medicamentos (CEI/CEIm), ensuring compliance with standard research integrity and participant safety requirements.

#### 3.5.10 Limitations and Future Directions

Measuring subjective experiences indirectly and ensuring complete unconsciousness under anesthesia presents methodological challenges. Future studies should consider large sample sizes, if necessary, and alternative methods for objectively assessing subjective experiences or unconscious psychedelic states. Exploring different anesthetic agents, sedation states, or incorporating control conditions without psilocybin may enhance clarity regarding specific neural and experiential effects. Future studies could also investigate different dosages and long-term follow-up to assess durability of therapeutic effects. A key limitation of this study design is that (P) will receive integration psychotherapy sessions, whereas the (P+A) will not. This introduces a potential confound, as any observed differences in therapeutic outcomes between these groups may be partially or entirely attributable to the integration process rather than the pharmacological effects of psilocybin alone. Consequently, it remains unclear whether the therapeutic benefits arise from the psychedelic experience itself or from the structured psychological support provided postsession. Future research should address this issue by implementing study designs that isolate the specific contributions of integration therapy versus the direct neurochemical effects of psilocybin, such as including a nonpsychedelic control group that also receives integration therapy or employing alternative methodologies to systematically dissociate these factors.

### 3.6 Animal Studies

The experimental design proposed here, comprising three distinct groups (e.g., Psilocybin Only (P), Psilocybin plus anesthesia (P+A), and anesthesia only (X)), could be adapted effectively for animal models, particularly in rodent studies involving conditioned fear paradigms. Employing a rodent model would enable a precise manipulation and measurement of memory reconsolidation, providing complementary evidence to human clinical trials. Specifically, such an approach could confirm whether memory reconsolidation and the therapeutic efficacy of psychedelics critically depend upon subjective experience. Furthermore, animal studies may provide additional mechanistic insights, as they allow for direct observation of behavioral, molecular, and neurophysiological changes that are less accessible in human studies. Translating our design into animal models could therefore offer convergent understanding about the causal importance of consciousness, or alternatively, suggest viable therapeutic pathways independent of subjective experience.

## 4 Discussion and Potential Implications

In case that the experimental results demonstrate that subjective experience is required for obtaining therapeutic benefits from psychedelic intervention, the following implications would emerge (see Table 2).

**Table 2:** Potential Outcomes and Related Implications

Implication Category	Same Therapeutic Effect Without Subjective Experience	No Therapeutic Effect Without Subjective Experience
Neurobiological Mechanisms	Therapeutic benefits are predominantly driven by direct neurobiological and brain connectivity changes.	Therapeutic benefits require the contribution of higher-order subjective processing.
Role of subjective Experience	Supports a more reductionist view where mental states are secondary to, or byproducts of, neurobiological activity.	Provides empirical support for the idea that mental states are causally efficacious, validating top-down causation models.
Clinical Treatment Protocols	Could lead to a revaluation of treatment protocols by suggesting that the intensity or quality of the subjective experience is not a critical parameter, potentially allowing for more flexibility in how treatments are administered. Could justify the development of nonpsychoactive analogues.	Would emphasize the need to create therapeutic settings that maximize and integrate meaningful subjective experiences, reinforcing the importance of “set and setting” as well as preparatory and integration sessions in clinical practice.
Philosophical Implications	Supports reductionist or physicalist views of consciousness.	Strengthens arguments for nonreductive physicalism or dual-aspect theories.
Future Research Directions	Encourages further investigation into the neurobiological underpinnings of psychedelics and their therapeutic effects.	Opens avenues for research that aims to dissect and optimize the qualitative aspects of the psychedelic experience, encouraging studies that explore which facets of the subjective state (e.g., insight, emotional release) are most therapeutically beneficial.

### 4.1 Clinical Implications

On the one hand, the findings would reinforce the notion that the psychological, subjective effects of psychedelics are not mere (and undesirable) side effects but are integral to the therapeutic process, which could have relevance in the development of new compounds.^48^ On the other hand, clinical protocols for psychedelic-assisted therapies might need to be adjusted to maximize the quality and integration of the subjective experience. For example, treatment settings could be optimized to create a safe and supportive environment that encourages meaningful experiences.

### 4.2 Clinical Research Implications

These findings would open new avenues for research aimed at exploring the most therapeutically beneficial aspects of subjective experience. Future investigations could focus on characterizing the types, intensities, and durations of subjective phenomena that correlate with the most significant clinical improvements, ultimately leading to optimized dosing strategies and therapeutic interventions.

While similar research proposals have aimed at isolating therapeutic effects from psychedelic experiences by blocking specific receptor sites (e.g., administering psilocybin alongside risperidone),^28^ these approaches still involve subjective conscious experiences, albeit modified or attenuated. In contrast, our proposed design using general anesthesia represents a more rigorous test of the necessity of subjective experience, as it completely eliminates consciousness during psychedelic intoxication. This allows us to robustly evaluate whether therapeutic effects can occur independently from any form of subjective awareness, thereby providing direct evidence on the role consciousness plays in psychedelic-assisted therapy.

### 4.3 Methodological Implications

Conducting double-blind randomized controlled trials (RCTs) with psychedelics presents significant challenges due to the psychoactive effects induced by these substances, which can lead to functional unblinding,^49^ as both participants and researchers often become aware of the treatment allocation, thus potentially introducing expectancy biases that can confound study outcomes. To address this issue, our study incorporates an anesthesia-plus-placebo (X+A) group, where participants receive general anesthesia and a placebo instead of psilocybin. This novel approach enhances the integrity of the double-blind methodology by ensuring that neither participants nor researchers can distinguish between the psilocybin-plus-anesthesia (P+A) and anesthesia-plus-placebo (X+A) groups based on subjective experiences during the intervention. By effectively masking treatment conditions, this approach minimizes expectancy effects and strengthens the validity of the study’s findings regarding the role of conscious experience in psychedelic therapy.

### 4.4 Broader Implications

Therapeutic change only in the presence of conscious experience would also support the idea that subjective experiences are not mere epiphenomena but have a direct, causal role in neural and neuropathological states. This finding would challenge reductionist views that consider neural processes as solely responsible for psychological outcomes and bolsters arguments for nonreductive physicalism or dual-aspect theories, where both neural and experiential aspects contribute to mental phenomena. The necessity of subjective experience would provide strong empirical support for top-down causation models, where higher-level mental states (e.g., the qualitative, subjective aspects of a psychedelic experience) exert influence on lower-level neural processes, potentially altering brain connectivity and therefore promoting enduring therapeutic effects, challenging strictly bottom-up approaches in neuroscience. Even further, demonstrating that subjective experience has a tangible impact on clinical outcomes could lend weight to arguments that the qualitative aspects of consciousness (“what it is like” component) are integral to understanding the mind-body relationship, thus encouraging further interdisciplinary research aimed at bridging the explanatory gap between neural mechanisms and subjective experience, thereby advancing theories that seek to resolve the so-called “hard problem” of consciousness and pushing a reevaluation of existing models of mental causation by suggesting that mental causation is not an illusion but a real phenomenon that can mediate long-term behavioral and cognitive transformations. Reinforcing the significance of subjective experiences in our understanding of the mind and mental health could also shift the focus of consciousness studies toward a more holistic view that accounts for both the physical substrates and the qualitative aspects of mental life, potentially inspiring new frameworks in cognitive science, psychiatry, and even artificial intelligence.

## 5 Conclusion

This proposal emphasizes the importance of thoroughly investigating the role of subjective experiences in psychedelic-assisted therapy. The proposed experimental design, employing general anesthesia with propofol, represents a novel approach to differentiate subjective experiences from purely neurobiological actions of psilocybin. The potential outcomes from this experiment could substantially clarify whether subjective consciousness is essential for therapeutic efficacy. Confirming this necessity could strengthen arguments for integrating subjective experiences into clinical protocols and challenge overly reductionist views of consciousness. Conversely, finding therapeutic effects independent of subjective experience might contribute to the development of new strategies of psychedelic therapy at a theoretical and practical level. Ultimately, this study aims to foster deeper interdisciplinary exploration, bridging gaps between neuroscience, psychiatry, and philosophy, and enriching our broader understanding of consciousness, mental health, and therapeutic mechanisms.
